# Effectiveness of a medication-adherence tool: study protocol for a randomized controlled trial

**DOI:** 10.1186/s13063-016-1393-2

**Published:** 2016-06-03

**Authors:** Mirrian Hilbink, Joyca Lacroix, Linda Bremer - van der Heiden, Aart van Halteren, Martina Teichert, Jan van Lieshout

**Affiliations:** Radboud University Medical Center, Radboud Institute for Health Sciences, IQ healthcare, PO box 9101, Code: 114 IQ healthcare, 6500 HB Nijmegen, The Netherlands; Behavior, Cognition & Perception, Philips Research, Eindhoven, The Netherlands

**Keywords:** Medication adherence, Barriers, Primary care, Cardiovascular diseases, Diabetes

## Abstract

**Background:**

Research shows that more than half of the people taking medication for a chronic condition are non-adherent. Nonadherence hinders disease control with a burden on patient quality of life and healthcare systems. We developed a tool that provides insight into nonadherence risks and barriers for medication-adherence including an intervention strategy to overcome those barriers. This study aims to assess the effectiveness of using this adherence tool in starters with cardiovascular or oral blood glucose-lowering medication to improve medication-adherence.

**Methods/design:**

In a cluster-randomized controlled trial 25 pharmacies in the Netherlands will be randomized to the intervention or control arm. Patients registered in a general practice participating in a collaborative can be included when they start cardiovascular or oral blood glucose-lowering medication prescribed by their general practitioner.

Participants complete an assessment consisting of measuring nonadherence risk and potential barriers to adherence. For patients with an increased nonadherence risk, a graphic barrier profile is created, showing to what extent eight cognitive, emotional, or practical barriers are present.

All patients will fill in the medication-adherence assessment twice: between 1 and 2 weeks after the start of the medication and after 8 months.

The intervention strategy consists of discussing this barrier profile to overcome barriers. Pharmacists and assistants of the intervention pharmacies are trained in discussing the profile and to offer a tailored intervention to overcome barriers. In the control arm, patients receive care as usual.

The primary outcome is medication-adherence of patients with a high risk of nonadherence at 8 months follow-up.

Secondary outcomes include the difference in the percentage of patients with an increased nonadherence risk between intervention and control group after 8 months, the predictive values of the baseline questionnaire in the control group in relation to medication-adherence after 8 months, medication-adherence after 1 year follow-up, and barriers and facilitators in the implementation of the tool.

**Discussion:**

This manuscript presents the protocol for a cluster-randomized clinical trial on the use of an adherence tool to improve medication-adherence. This study will provide insight into the effectiveness of the tool in starters with cardiovascular or oral blood glucose-lowering medication in improvement of medication-adherence.

**Trial registration:**

The Netherlands National Trial Register, NTR5186. Registered on 18 May 2015.

**Electronic supplementary material:**

The online version of this article (doi:10.1186/s13063-016-1393-2) contains supplementary material, which is available to authorized users.

## Background

Adherence to chronic medication is problematic in clinical practice. Nonadherence leads to poor disease control with a burden on patient quality of life and healthcare systems [1]. Research shows that, on average, 50 % of patients with a chronic condition are not adherent, with adherence estimates ranging from 17 to 80 % [2–4]. Drugs for asymptomatic chronic conditions are found to have especially low adherence rates [5]. In several studies, the risk for nonadherence was shown to be highest in the first year after the start with chronic medication [6, 7]. Consequently, interventions to warrant adherence are expected to be most effective at the initiation of a chronic medication treatment.

Various causes have been demonstrated to hamper adherence [4, 8]. Conventional models distinguish between intentional and nonintentional barriers as causes for poor adherence [9]. Intentional barriers develop because of patients’ beliefs and perceptions about their medications and diseases. These barriers can be further subdivided into cognitive and emotional barriers. Nonintentional barriers depend on capacity, resources, and practical barriers [10]. Besides personal beliefs, adherence depends on the type of disease but may also vary within patients over time [10]. The multifaceted nature of the adherence problem illustrates that improving adherence is complex and needs interventions tailored to the individual patient [9]. A recent Cochrane review showed that current methods of improving medication adherence for chronic health problems are mostly complex and not very effective [11]. The effectiveness of nonadherence interventions can be improved by targeting the underlying barriers related to nonadherence for a specific patient. Interventions can focus on cognitive and emotional barriers (intentional nonadherence) or on practical barriers (nonintentional nonadherence), each with their own specific intervention ingredients tailored to patients’ needs. Depending on the character of the intervention, different primary healthcare providers can be involved. For instance, the pharmacist may be better equipped to implement aids to overcome practical barriers, and general practitioners (GPs) and nurses may be better able to help the patient overcome cognitive and emotional barriers. Though there are several instruments assessing actual medication adherence and various programs to improve adherence, at present, no tool is available for healthcare providers to predict the risk and potential causes for nonadherence at the start of chronic medication with a tailored approach to overcome the individual barriers detected. Such a tool would enable them to address potential barriers to nonadherence and prevent early cessation of chronic medication. Therefore, a predictive tool is needed at the start of chronic medication to assess the risk of future nonadherence and to address the assessed causes of the nonadherence for a patient at high risk.

Furthermore, an implementation strategy is needed to provide tailored interventions in primary healthcare settings. For the efficient implementation of effective interventions in existing primary healthcare workflows, a good cooperation between pharmacists, GPs, practice nurses, and assistants is required. Technology-based systems that enable easy communication between healthcare providers about the progress of interventions and agreement about the allocation of time and resources need to be in place.

At Philips Research, a questionnaire was developed that creates a barrier profile based on an online assessment. The questionnaire consists of two parts: the Probabilistic Medication Adherence Scale (ProMAS), which is an 18-item questionnaire to assess nonadherence risk [12], and an additional 23 questions that assess the underlying barriers related to nonadherence. The resulting barrier profile provides insight into the extent to which eight cognitive, emotional, and practical barriers may hamper a patient’s adherence. For each of the identified barriers, a set of recommendations was formulated in a manual for healthcare providers on how to address the barriers in an intervention. Consequently, in patients starting chronic medication and at high risk for nonadherence, as assessed by ProMAS, the barrier profile provides an intervention strategy to overcome cognitive, emotional, and practical barriers to adherence at an early moment and thereby promotes adherence.

The primary objective of this study is to assess the effectiveness of using the medication-adherence tool in starters with cardiovascular medication, which includes antiplatelets, antihypertensives, and lipid-lowering drugs (Appendix), and oral blood glucose-lowering medication in the improvement of medication adherence as measured by pharmacy dispensing data. A second objective is to evaluate the implementation strategy to reduce barriers identified in patients at risk for nonadherence for a primary care setting relating to the acceptability of the workflow for all stakeholders (pharmacists, general practitioners, and patients).

The primary research question is as follows:

1. What is the benefit of using the medication-adherence tool for medication adherence of patients starting with cardiovascular or oral blood glucose-lowering medication identified as being at high risk for nonadherence at a follow-up of 8 months compared to usual care?

Secondary questions are as follows:

a) What is the association between the ProMAS score at 8 months and the adherence after 8 months?
  b) To what extent can the identified barriers at 8 months predict medication adherence after 8 months?
What are the predictive values of the ProMAS score and the barrier profile, measured at drug initiation, in relation to medication adherence after 8 months for all patients in the control group who started with cardiovascular of diabetes medication?
What is the adherence of the subgroup of patients with a longer follow-up period of at least 1 year after drug initiation?

Process evaluations will address the following question:

1. What are the barriers and facilitators, as experienced by pharmacists and patients, regarding the implementation of the medication-adherence tool in their daily practice?

The aim of this study protocol is to describe the design and methods of a study to assess the effectiveness of using a medication-adherence tool in starters with cardiovascular or oral blood glucose-lowering medication to improve medication adherence.

## Methods/design

### Study design

This study is a cluster-randomized trial with an intervention group of pharmacies (using the adherence tool) and a control group of pharmacies (providing usual care). The key components of the medication-adherence tool are described in Table 1.

**Table 1:** Components of the medication-adherence tool

*■* Online questionnaire consisting of two parts
a) the Probabilistic Medication Adherence Scale (ProMAS), slightly adapted for use with patients with a first prescription of cardiovascular or oral blood glucose-lowering medication
b) assessment of the underlying barriers related to nonadherence
*■* Tools used by pharmacists to facilitate the provision of a tailored intervention
a) a 3-hour training session on patient-centered communication and discussion of the barrier profile
b) manual containing instructions for discussing and overcoming the various potential barriers for medication adherence
c) an internet-connected tablet to register new participants, to review the graphical barrier profile at the time of the second dispensing, and to make any notes regarding the conversation and applied intervention
*■* Tailored intervention

As the second dispensing, a tailored intervention is initiated for patients in the intervention group with an increased risk for nonadherence. All interventions start with a discussion of the patient’s barrier profile. The intervention will be tailored to the identified emotional, cognitive, and practical barriers of the patient.

As in daily practice, patients receive a new medication from their community pharmacist; therefore, the assessment of patients’ medication adherence and potential barriers was most appropriately situated in the community pharmacy for our study. In the Netherlands, patients with a first dispensing of a chronic drug usually receive only an amount for 2 weeks. Treatment is continued after 2 weeks according to the experiences of the patient. Thus, patients starting with the study medication will have to have contact with the community pharmacy within approximately 2 weeks. This makes it convenient to ask eligible patients to participate in the study at the first dispensing, let them fill in the medication adherence assessment before the second dispensing, and apply the intervention when necessary at the second dispensing. The feasibility of implementing the use of the adherence tool in the daily pharmacy workflow was piloted in two pharmacies. During the pilot period, researchers regularly contacted the pilot pharmacists in order to track their experiences and any developments and to solve any issues. After the pilot period, a meeting between the research team and the two pilot pharmacists was planned for evaluation of the pilot. Based on the experiences during the pilot period, workflow adaptations leading to an increase in convenience and efficiency were made. Small changes in study information for patients, pharmacists, and GPs were made after the pilot period.

**Fig. 1:**
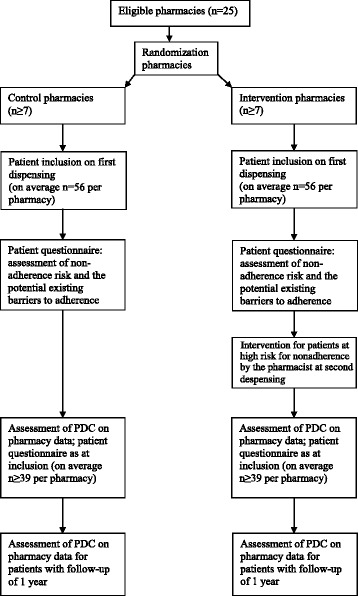
Flow diagram of the study

For further clarification of this study protocol, we reference the study flow diagram (Fig. 1) and the SPIRIT checklist (Additional file 1).

### Recruitment of pharmacies, general practices, and patients

The trial will be carried out with pharmacists, general practitioners, and their patients with a first prescription of cardiovascular medication, including antihypertensives, antiplatelets, and lipid-lowering drugs, or oral blood glucose-lowering medication made by their GP within the study period (see Appendix for drug classes and ATC codes). A first prescription is defined as no drug dispensing from a drug class issued to the patient in the preceding year. For example, a patient who used a beta-blocking agent in the preceding year is not eligible when starting another beta blocker but will be eligible when starting a statin.

The primary care cooperation De Ondernemende Huisarts (DOH), or The Innovative General Practitioner, will play a key role in the recruitment of pharmacies and general practices for this study. DOH is a general practice collaborative, i.e., approximately 70 GPs working in 16 group practices in the southeastern part of the Netherlands who that share an overarching management structure and develop and implement structured care for several prevalent chronic conditions, including cardiovascular diseases and diabetes.

#### Pharmacies

The DOH cooperates closely with a number of community pharmacies (so-called CaZo cooperation). In total, 25 pharmacies have joined the cooperative. A representative of DOH will contact all 25 pharmacies connected to the DOH cooperation and provide them an invitation and an information leaflet about the study. Three members of the research team will visit all pharmacists who express their intention to participate. After a detailed explanation of the study aims, methods, and expected outcomes to the pharmacists, written informed consent for both participation and collection of de-identified patient data from the pharmacist’s database will be obtained.

The participating pharmacies are randomized for the intervention or control group, which implies that the patients are clustered within pharmacies. Pharmacies are stratified into small or large depending on their number of patients registered with DOH GPs. After the randomization procedure (drawing from a hat by an independent research assistant and consecutive allocation) has been performed, the pharmacies will be informed of their assignment to either the intervention group or the control group. Only pharmacists and pharmacy assistants from the intervention group are being trained to interpret the barriers to adherence profile and to provide tailored care. This prevents contamination between the intervention and control patients. Because patients in the Netherlands predominantly visit one pharmacy for all their medication, pharmacists are assumed to have complete medication dispensing information for their patients [13].

Due to the test of the medication-adherence tool, both pilot pharmacies will have to participate in the intervention group.

#### General practices

Participating DOH general practices will receive written information on the study. GPs will be asked to give written informed consent to participate in the study and to share patient information from their electronic medical record system They will be encouraged to contact a member of the research team with their questions if anything is unclear.

#### Patients

##### Inclusion criteria

Patients will be eligible for the trial if all the following criteria are met: age > 18 years, being prescribed a cardiovascular or glucose-lowering drug for the first time by a DOH GP. The pharmacist will be signaled by his computer system when a patient visits with a first prescription.

##### Exclusion criteria

Patients will be excluded if they have any cognitive impairments or insufficient Dutch language skills as determined by the pharmacists' judgment. Inclusion numbers will be monitored; inclusion will be stopped when the targeted number is reached.

### Outline of the study procedure for patients

For all patients starting with cardiovascular or oral blood glucose-lowering medication with a first prescription from a DOH GP, the GP starts a treatment plan in the electronic patient information system that the GPs and pharmacies use in their current daily practice. Then, the following process starts when the patient comes to the pharmacy for the first dispense.

1. On first dispensing at the pharmacy, when the pharmacist considers the patient eligible for participation, the patient receives a short oral introduction to the study and an invitation to participate in the study.
2. When the patient is interested in participation, the pharmacist asks for the patient’s permission to send the patient an email with a link to the study survey and collects the patient’s Citizen Service Number (CSN). Additionally, the pharmacist makes an appointment for the second dispense.
3. The pharmacist (assistant) uses a tablet/web browser to register the patient for potential enrollment in the study.
4. One week after the pharmacy visit, the computer system sends an email to the patient containing the link to the questionnaire.
5. Then, the patient receives a paper-based package of study information and consent form to take home. This information will be provided on top of the usual information given during the first dispense in the pharmacy and will not replace any information that is usually provided during the first dispense.
6. At home, the patient reads the study information and decides on participation. If the patient decides to participate, (s)he answers the link in the email received and completes the survey. The answers are used to produce a profile, which is sent to the electronic patient file system so that the pharmacists of the intervention practices have it available during the second dispensing appointment when the intervention starts (For control patients, the profile is only made available after completion of the study). The survey answers are retained until the end of the study.
7. The patient sends the signed informed consent forms by regular mail in a pre-stamped envelope.
  For the second dispensing at the pharmacy, the pharmacists in the control arm do not receive the results of the medication adherence assessment from their patients, and they provide usual care. This includes the usual information at the start of a new drug as described in the guidelines of the pharmacists’ profession (KNMP richtlijn ‘ter hand stellen’, http://www.knmp.nl/praktijkvoering/bekostiging/begeleidingsgesprek-nieuw-geneesmiddel/knmp-richtlijn-ter-hand-stellen-juni-2013).
8. The pharmacist in the intervention group checks whether the patient has completed the medication adherence assessment and takes care to provide a second dispensing of the drug.
9. If the patient has not completed the assessment at the moment of the second dispensing, the patient is asked whether (s)he would like to participate and is asked to complete the survey in the pharmacy on a tablet.

Patients that prefer to participate with a paper-based questionnaire instead of an online questionnaire can sign and send in the paper informed consent form and indicate their preference. They will receive the questionnaire per regular mail together with a return envelope. Those participants that fill out a paper-based questionnaire return the questionnaire by regular mail, and their data will be entered into the system by a research worker. As an alternative, participants who prefer the online questionnaire but do not have access to a computer or Internet at home can fill out the questionnaire at the pharmacy during their visit for the second dispensing.

At the second dispensing moment, pharmacists in the intervention group will initiate a tailored intervention for patients with an increased risk for nonadherence. The intervention will be tailored to the patient’s emotional, cognitive, and practical barriers, as identified by the barriers assessment. Patients without an increased risk for nonadherence, receive usual care (delivery of medication only). As mentioned above, the ProMAS scores and profiles will be stored in the electronic patient information system.

### The intervention

**Fig. 2:**
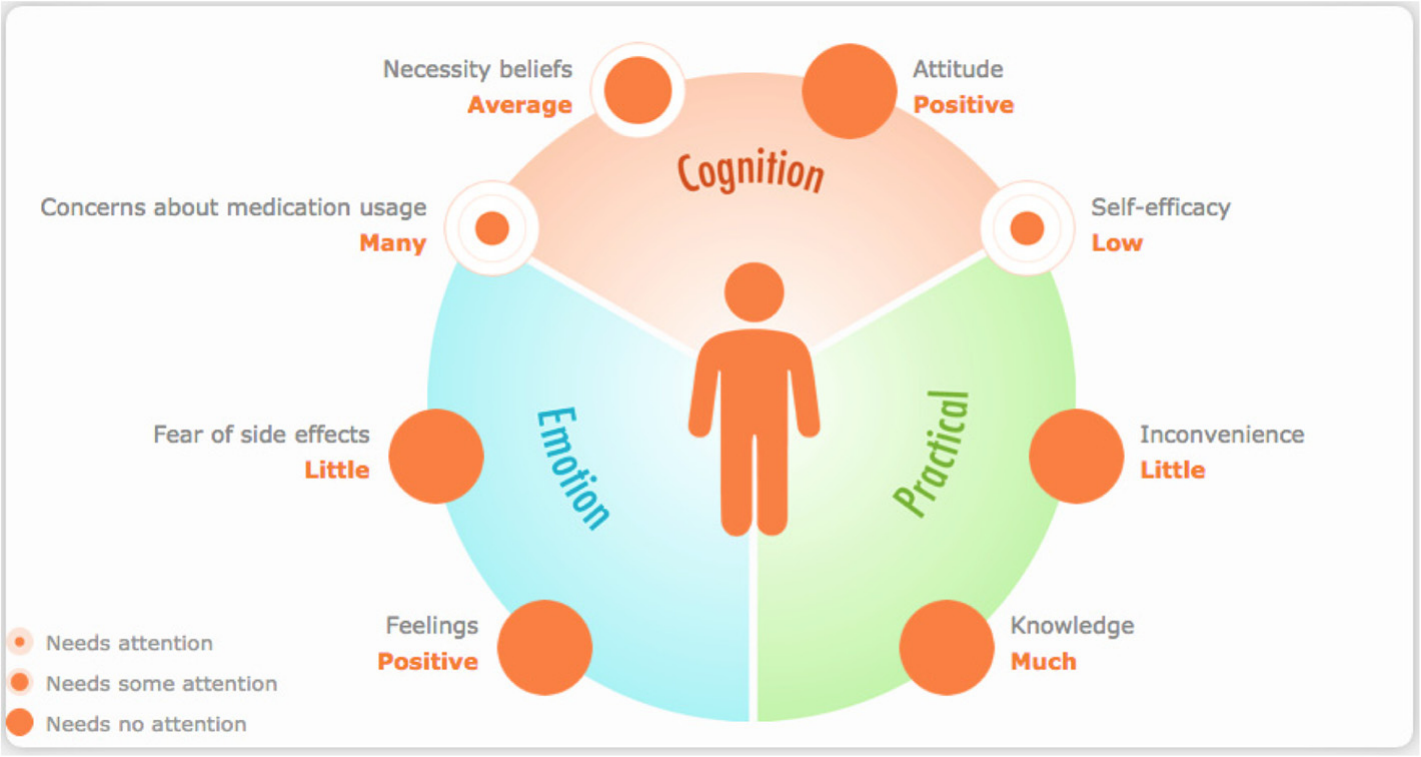
Example of a barrier profile. The profile shows eight potential adherence barriers: two emotional barriers (feelings with regard to medication and fear of side effects); one emotional/cognitive barrier (concerns about medication usage); two cognitive barriers (necessity beliefs and attitude with regard to medication); one cognitive/practical barrier (self-efficacy), and two practical barriers (inconvenience and knowledge about medication scheme)

In the pharmacy, the pharmacist will intervene at the second dispensing for those patients with a PROMAS score that signals an increased nonadherence risk. In the intervention pharmacies, the barrier profile (see Fig. 2), in combination with the manual, will be used as a systematic decision support tool to tailor interventions to the identified cognitive, emotional, and practical adherence barriers. The manual serves as a guideline for discussing the identified barriers and determining an intervention to address them. Table 2 describes two examples of barriers with recommendations from the manual on how to address these barriers and to choose an intervention. In the barrier profile, the extent to which each of the eight potential barriers are present will be depicted graphically in three gradations (see Fig. 2).

**Table 2:** Examples of recommendations on how to address the barriers in an intervention

*■* In case “fear of side effects” seems to be a barrier
○ Ask questions to try to find the cause(s) for de fear of side effects and identify the influence of external factors such as media and other persons.
○ Focus on the elimination of the fear, using the following possibilities:
- explain that the medication can cause side effects and provide the patient information about the “number needed to harm,
- discuss the balance between the risk for side effects and the desired effect,
- discuss the best way for medication intake to prevent side effects, and
- agree about the intake of the medication and encourage the patient to report existing side effects.
Examples of questions:
○ How do you generally think about the fact that you need to take medication?
○ What is your attitude toward medication, and toward the prescribed medication in particular?
○ What information did you get about the benefits of the medication?
○ For which side effects do you have fear and where does it come from?
○ Did this fear always exist or did it change? What caused this change?
○ How does this fear influence you? What is the effect on your medication intake?
○ Do you have information on your risk for particular side effects? ○ Which side effects play a major role in your well-considered decision on the intake of this medication?
○ Which side effects do you expect, and to what extent do they prevent you from medication intake?
○ Let’s go through the information leaflet to discover which side effects can possibly occur in your case.
*■* In case “knowledge about medication scheme” seems to be a barrier:
○ Discuss the prescribed use of the new medication.
○ Discuss the use of other chronic medication and assimilate the new medication into the patient’s medication scheme.
○ Take the patient’s customs into account.
○ Explain the importance of regular medication intake for the efficacy of the medication and the possible side effects and interaction with other medication.
○ Choose the most appropriate option for the patient:
- consider whether medication synchronization or a repeat prescription service will be helpful for the patient,
- consider reminders for medication intake,
- consider the use of a pill box at the start of each week with all medications,
- consider streamlining medication regimens and the development of a new treatment plan, and
- consider to involve an informal caregiver.
Examples of questions:
○ Do you have sufficient knowledge about your new medication scheme including all your medication?
○ Is this medication scheme feasible for you?
○ Do you need any support with regard to your medication intake?

All interventions start with a discussion of the barrier profile with the patient in a manner that makes use of the patient-centered techniques. When cognitive or emotional barriers (e.g., attitude with regard to medication, concerns about medication usage, and fear of side effects) are present for the specific patient, the pharmacist starts to discuss the relevant issues in the profile using the recommendations of the manual and the patient-centered communication techniques to try to take away or diminish these barriers. The manual proposes the following main intervention strategies: for patients with an insufficient awareness of the need to use the medication, self-assessment of clinical measures (blood pressure and blood glucose) will be proposed, since this has been shown to increase medication adherence. When agreed on by the patient, this will be initiated in the pharmacy by providing the patient the relevant devices. When barriers mainly appear to be practical for the specific patient (inconvenience and/or knowledge about medication scheme), the pharmacist offers one or several practical solutions, e.g., suggestion for medication change, pill organizer, use of a Baxter system, reminders, seek the help of the patient’s social network, or simplification of dosage scheme. The pharmacist registers the intervention.

Since the pharmacist as well as the GP and the nurse practitioner are involved in supporting their patient with their medication use, a good communication and cooperation among all these parties is needed.

Technology-based systems that enable easy communication between healthcare providers about the progress of interventions and agreement about allocation of time and resources are required for optimal functioning of the medication-adherence tool. The chain information system Care2U is the preferred way to communicate all information about the interventions. Currently, the system needs some adaptations in order to enable such communication between pharmacy and general practice. In case such communication is not possible by the time of the start of the study, other ways of communication (telephone, email, or fax) between pharmacists and GPs will be agreed upon.

### Implementation of the medication-adherence tool

To deliver the tailored intervention, training on a patient-centered communication technique will be offered to the pharmacists, and one of the pharmacy assistants of the pharmacies assigned to the intervention group. Intervention pharmacists and their pharmacy assistants will receive a three-hour training session before starting the trial, consisting of a brief introduction to the proposed communication technique, demonstration of a second dispense moment with a barrier profile, and skills practice using role play. The pharmacists and pharmacy assistants will be specifically instructed on discussing the profile with the patients in order to offer a tailored intervention to overcome cognitive, emotional, or practical barriers.

Intervention pharmacies will also receive a manual containing instructions for discussing and overcoming the various potential barriers for medication adherence. In this manual, potential barriers are categorized into cognitive, emotional and practical nonadherence barriers. Furthermore, intervention pharmacies will receive a follow-up group session from the trainer on the patient-centered communication technique to provide support and exchange experiences on discussing the barrier profiles. In addition, three members of the research team will visit all participating pharmacists. At least the pharmacist and one experienced pharmacy assistant of a pharmacy will be present during that appointment. We will explain the study in detail and provide them with an easy-to-understand explanation of the workflow concept and subsequently discuss how the workflow concept can be optimally tailored to existing routine work procedures in the pharmacy. One of the researchers will be available during working hours to answer questions arising from the study. Finally, pharmacists will be offered the opportunity to contact one of the pilot pharmacists to ask questions and advices about the study procedures. Table 3 summarizes the various elements of the implementation strategy.

**Table 3:** Elements of the implementation strategy

*■* Visit of the pharmacy by the research team to provide the manual and to tailor the workflow concept to existing work procedures in an optimal way
*■* Training session for pharmacists on patient-centered communication, using the manual and discussing the barrier profile
*■* Follow-up group session of the trainer on discussing the barrier profile
*■* Onsite support on the use of the tablet
*■* Availability of researcher for answering questions about the study
*■* Possibility to contact pilot pharmacist for questions and advice on study procedures

### Measures

#### ProMAS

The Probabilistic Medication Adherence Scale (ProMAS) is an 18-item validated questionnaire to assess nonadherence behavior in general [12]. For the purpose of this study, the questionnaire will be slightly adapted for use with patients who do not take any other chronic medication and who have a first prescription of cardiovascular or oral blood glucose-lowering medication. The cut-off value for being at risk for nonadherence is 13: patients with a score of ≥ 13 are considered to have an increased risk for nonadherence, whereas patients with a score below 13 are not at risk for nonadherence. The ProMAS will be applied at baseline and at 8 months follow-up.

#### Scale on underlying barriers related to nonadherence

This questionnaire measures to what extent the following emotional, and practical barriers for nonadherence are present: feelings with regard to medication, fear of side effects, concerns about medication usage, necessity beliefs, attitude with regard to medication, self-efficacy, inconvenience, and knowledge about medication scheme.

#### Adherence

Medication adherence is calculated as the percentage of days covered by medication (PDC) based on pharmacy dispensing of one drug group since the start of the therapy until the end of the study period. Patients with a PDC of at least 80 % are labeled as “sufficiently adherent.” PDCs are calculated per medication group (see Appendix), except for blood glucose lowering drugs. As for diabetes, often, more than one drug is used, and patients have to adhere to each drug from the subclass, e.g. to biguanides and sulfanilamide derivatives. Data on dispensing will be based on routine pharmacy data collection.

### Outcomes

The primary outcomes are as follows:

1. The primary outcome is the difference in the medication adherence of patients with an initial high risk of nonadherence between the intervention and the control group, measured by pharmacy dispensing records at 8 months follow-up.

The secondary outcomes are as follows:

1. Based on data from all patients participating:
  - The difference in the percentage of patients with an increased risk for nonadherence based on the ProMAS between the intervention and control group at 8 months follow-up
  - The difference in percentage of patients with an increased risk for nonadherence based on the barrier profile between the intervention and control group at 8 months follow-up
2. The positive and negative predictive values of the ProMAS score and the barrier profile measured at drug initiation in the control group in relation to medication adherence at 8 months follow-up
3. Difference in medication adherence in the subgroup of patients with a follow-up period of at least 1 year after drug initiation. This in fact means to assess an effect after a longer follow-up period of at least a year analogous to the primary outcome.

The process evaluation will address the following:

1. The barriers and facilitators in the implementation of the use of the medication-adherence tool.

These influencing factors will be discussed with all pharmacists in the intervention arm by semistructured interviews. The assessment on barriers and facilitators will be based on the Tailored Implementation for Chronic Diseases’ (TICD) checklist. This is a comprehensive, integrated checklist with 57 potential determinants of practice grouped in seven domains: guideline factors, individual health professional factors; patient factors; professional interactions; incentives and resources; capacity for organizational change; and social, political, and legal factors [14].

Patients will also be interviewed about their experiences with the medication adherence assessment and interventions. Informed consent for these interviews will be obtained. Interviews will aim at gaining a better understanding of how patients and healthcare providers have experienced the intervention in terms of effort, time investment, and benefits. The purposive patient sample size will be determined based on theoretical saturation (data collection until no new information is obtained). Therefore, data review and analysis will be done in conjunction with data collection.

### Sample size

For the sample size calculation, the following assumptions are made: we assume that a percentage of 60 % is nonadherent in our sample of patients at high risk based on their ProMAS score. This assumption is reasonable according to published studies [6]. We assume that the intervention will increase the 80 % PDC in the intervention group by 20 % compared to the control group. As patients are clustered within pharmacies, the evaluation will be performed by multilevel analysis to adjust for clustering. For the effect of clustering, an intracluster correlation (ICC) of 0.05 was assumed.

In agreement with conventional power calculations, a power of 80 % to detect a true difference and a chance of 5 % of a type 1 error to wrongly conclude on a difference are assumed. For this trial, in the DOH/CAZO setting, we assume that there are at least 14 community pharmacies willing to participate. The sample size calculation results in 39 patients needed per pharmacy. PASS software version 11 was used to determine the appropriate sample size. Considering 30 % lost to follow-up during the study period, 56 patients need to be included per pharmacy to achieve the power needed. If only 50 % of the patients agree to join the study, 112 patients need to be invited per pharmacy.

In the potentially participating 25 pharmacies, a total of at least 6,081 single patients with primary dispensings of cardiovascular or oral blood glucose-lowering medications were detected within 12 months (as observed in historical data). This means that, on average, 20 subjects started with cardiovascular or oral blood glucose-lowering medication monthly per pharmacy. Assuming 40 % of the starters at risk for nonadherence (determined by a ProMAS score ≥ 13), this means, on average, that eight patients are available monthly per pharmacy for an intervention. The period to have 112 starters on cardiovascular or blood glucose-lowering medication per pharmacy will be approximately 14 months.

A measurement period of at least 8 months is preferred in order to get valid information from dispensing data. A first prescription is usually dispensed for 14 days, followed by a prescription for 90 days. After 8 months, at least two repeat prescriptions after the second dispensing must have had occurred to meet a PDC of 100 %. As we assume a PDC of 80 % is sufficient, at least (14 days + 180 days =) 196 days * 1.2 = 234 days = 7.8 months are needed to detect nonadherence. If the last patient was included after 14 months, together with the measurement period, this ends up the total study period with 22 months needed for follow up.

### Data analysis

The primary analysis will be an intention-to-treat analysis to test whether patients starting with cardiovascular or oral blood glucose-lowering medication who are at high risk for nonadherence and who received a tailored intervention based on the patient’s identified emotional, cognitive, and practical barriers will be more adherent compared to patients who did not receive the intervention, expressed as PDC. We will compare differences in medication adherence between the intervention and control group both as a dichotomous (PDC < 80 % versus PDC ≥ 80 %) and continuous outcome measure (mean PDC). Because patients are clustered in pharmacies, we will perform multilevel analyses, with adjustment for potential confounders (patient age, gender, diagnosis, and number of medications). Secondary analyses will test the hypothesis that patients who received the tailored adherence intervention will be more adherent compared to patients who did not receive this intervention, expressed by the ProMAS score. We will compare differences in the ProMAS score between intervention and control group both as a dichotomous (score < 13 versus ≥ 13) and continuous outcome measure (mean score). To assess differences between groups, we will perform linear and logistic mixed models. Further secondary analyses will assess the frequency of different barriers for medication adherence. We will compare differences in the profile score between intervention and control group both as a dichotomous (per barrier, present, or absent) and continuous (mean number of barriers present) outcome measure. Finally, the predictive values of the ProMAS score and the score on the eight identified barriers on patient adherence, based on dispensing data, will be quantitatively assessed. All statistical analyses will be performed using SPSS software (version 20, IBM Corp.) or MLWIN (version 2.28, University of Bristol).

We will qualitatively analyze the interviews with pharmacists and patients. The interviews will be transcribed verbatim. The transcripts will be analyzed by open coding at macro level using predefined main codes. After the open coding, the research team will group themes and subthemes for each level of healthcare. The program Atlas.ti (version 6, ATLAS.ti Scientific Software Development GmbH) will be used for analysis.

### Steering committee

Five of the authors (JL, LB-vdH, AvH, MT, and JvL) together with a project leader from DOH and a pharmacist form the steering committee. In weekly telephone conferences, they closely follow the development of the study with the possibility to make adjustments when necessary.

## Discussion

This manuscript presents the protocol for a cluster-randomized clinical trial on the effectiveness of an adherence tool to improve medication adherence in starters with cardiovascular or oral blood glucose-lowering medication. A trial provides an optimal design to elucidate the effectiveness of interventions tailored to the underlying cognitive, emotional, and practical barriers to nonadherence identified for the patient. The results will provide insight in both the effectiveness of applying the medication-adherence tool and the predicting value of the ProMAS and the identified barriers for medication nonadherence. The effect of interventions to increase adherence depends on many factors. However, many approaches to improve adherence apply the one-size-fits-all-principle. By discussing the barrier profile with the patient, pharmacists can provide personalized support. An important and unique component of this study is the provision of an intervention tailored to the patient’s emotional, cognitive, and practical barriers. Not only the intervention itself, but also the implementation of the intervention will be tailored. The research team will visit all participating pharmacies to tailor the workflow concept as much as possible to existing work procedures. As the information on the barriers and the intervention is shared among the healthcare providers, all information supplied to the patient is consistent. A limitation of our study is that the questionnaire on barriers related to nonadherence has not been extensively used and validated in earlier studies. Furthermore, randomization occurs at the pharmacy level. Therefore, GPs may have patients in both the intervention and control group, which could lead to contamination. However, the barriers are discussed with the patients in the intervention pharmacies only. As the GPs will only follow a part of the intervention from the implementation guide for those patients that are announced by the intervention pharmacists, this limitation is presumably not severe. This study will be carried out in the DOH/CAZO care groups. These care groups have innovative mindsets. Consequently, the implementation of the intervention in other care groups might need additional guidance. Within these care groups, recruitment bias may be caused by the response of patients with certain characteristics, including medication adherence. Due to privacy legislation, the characteristics of nonresponders will not be analyzed.

## Trial status

At the time of submission of this study protocol, we are recruiting pharmacies and patients for the trial.

## Abbreviations

CSN, Citizen Service Number; DOH: De Ondernemende Huisarts; GP, general practitioner; ICC, intracluster correlation; PDC, percentage of days covered; ProMAS, Probabilistic Medication Adherence Scale; TICD: Tailored Implementation for Chronic Diseases.

## Appendix

### ATC codes for cardiovascular or oral blood glucose-lowering medication

- ATC code A10B: oral antihyperglycemic drugs
- ATC code B01AC: platelet aggregation inhibitors
- ATC code C01A: cardiac glycosides
- ATC code C01D: vasodilators
- ATC code C03: diuretic drugs
- ATC code C07: beta-blocking agents
- ATC code C08: calcium channel blockers
- ATC code C09: agents acting on the renin-angiotensin system
- ATC code C10: lipid-lowering drugs

## Additional file

Additional file 1: SPIRIT checklist. (DOC 121 kb)

## References

[1] Schroeder K, Fahey T, Ebrahim S. Interventions for improving adherence to treatment in patients with high blood pressure in ambulatory settings. Cochrane Database Syst Rev. 2004. http://summaries.cochrane.org/CD004804/what-interventions-improve-adherence-to-treatment-in-patients-with-high-blood-pressure-in-ambulatory-settings#sthash.PHlaJ84Q.dpuf. Accessed 25 May 2016. 10.1002/14651858.CD004804 PMC9036187 15106262

[2] Dolce J, Crisp C, Manzella B, Richards J, Hardin J, Bailey W (1991). Medication adherence patterns in chronic obstructive pulmonary disease. Chest.

[3] Haynes RB, Ackloo E, Sahota N, McDonald HP, Yao X (2008). Interventions for enhancing medication adherence. Cochrane Database Syst Rev.

[4] Krueger KP, Berger BA, Felkey B (2005). Medication adherence and persistence: a comprehensive review. Adv Ther.

[5] Blaschke TF, Osterberg L, Vrijens B, Urquhart J (2012). Adherence to medications: insights arising from studies on the unreliable link between prescribed and actual drug dosing histories. Annu Rev Pharmacol Toxicol.

[6] Erkens J, Panneman M, Klungel O, Van den Boom G, Prescott M, Herings RM (2005). Differences in antihypertensive drug persistence associated with drug class and gender: a PHARMO study. Pharmacoepidemiol Drug Saf.

[7] Penning-van Beest F, van Herk-Sukel M, Gale R, Lammers JW, Herings R. Three-year dispensing patterns with long-acting inhaled drugs in COPD: a database analysis. Respir Med. 2011;105:259–65. 10.1016/j.rmed.2010.07.007 20705441

[8] Sarafino EP, Smith TW. Health interactions psychology: biopsychosocial. Chichester, West Sussex, PO19 8SQ, UK: John Wiley & Sons; 2010.

[9] Horne R (1999). Patient's beliefs about treatment: the hidden determinant of treatment outcome?. J Psychosom Res.

[10] Horne H, Chapman S, Parham R, Freemantle N, Forbes A, Cooper V. Understanding patients’ adherence-related beliefs about medicines prescribed for long-term conditions: a meta-analytic review of the necessity-concerns framework. PloS One. 2013. http://www.ncbi.nlm.nih.gov/pmc/articles/PMC3846635/pdf/pone.0080633.pdf. Accessed 25 May 2016. 10.1371/journal.pone.0080633 PMC3846635 24312488

[11] Nieuwlaat R, Wilczynski N, Navarro T, Hobson N, Jeffery R, Keepanasseril A (2014). Interventions for enhancing medication adherence. Cochrane Database Syst Rev.

[12] Kleppe M, Lacroix J, Ham J, Midden C (2015). The development of the ProMAS: a Probabilistic Medication Adherence Scale. Patient Prefer Adherence.

[13] Buurma H (2008). Prevalence and determinants of pharmacy shopping behaviour. J Clin Pharm Ther.

[14] Flottorp SA, Oxman AD, Krause J, Musila NR, Wensing M, Godycki-Cwirko M (2013). A checklist for identifying determinants of practice: a systematic review and synthesis of frameworks and taxonomies of factors that prevent or enable improvements in healthcare professional practice. Implement Sci.

